# Computational approaches for the identification of potential HDAC2 inhibitors and histamine H3 receptor antagonists from *Berberis vulgaris*: a dual mechanistic approach for autism spectrum disorder treatment

**DOI:** 10.1515/biol-2025-1272

**Published:** 2026-02-24

**Authors:** Tarique Sarwar, Hajed Obaid A Alharbi, Fahad A Alhumaydhi, Rashid Mumtaz Khan, Arshad Husain Rahmani

**Affiliations:** Department of Medical Laboratories, College of Applied Medical Sciences, Qassim University, Buraydah 51452, Saudi Arabia; Department of Chemistry, College of Sciences, Qassim University, Buraydah 51452, Saudi Arabia

**Keywords:** autism spectrum disorder, cinnamyl acetate, HDAC2, H3R, *Berberis vulgaris*, molecular dynamics simulation

## Abstract

Autism spectrum disorder (ASD) encompasses early emerging deficits in repetitive sensory-motor behaviors and social communication. Rooted in a solid genetic foundation, ASD exhibits diversity among individuals but consistently manifests core features in restricted repetitive behaviors and social communication. ASD originates from early neural reorganization and alterations in brain development, forming a spectrum from mild to severe. The economic burden of ASD is substantial, driven by the ongoing need for assistance in adulthood. Histone deacetylase (HDAC) modulation, including HDAC2, influences ASD traits. Interest in HDAC modulation has led to the FDA approval of drugs that show promise. Additionally, studies suggest histamine, a CNS neurotransmitter, and H3R antagonism may impact social behavior in ASD. Recognizing the significance of HDAC2 and H3R, we conducted virtual screening of phytocompounds from *Berberis vulgaris* against these ASD-associated targets. Cinnamyl acetate (CA) emerged as the primary compound for targeting ASD. Molecular dynamics simulations were conducted to explore the dynamics and stability. Of the tested compounds, only three exhibited AMES toxicity, and none were predicted to be hERG I inhibitors or to cause oral acute toxicity in rats. The interaction energies for CA docking to HDAC2 and H3R were −7.4 and −7.6 kcal/mol, respectively. The molecular dynamics simulation confirmed the stability of CA with target proteins under physiological conditions, revealing minimal perturbation to the proteins’ secondary structure upon CA binding. These findings underscore the potential of CA in the treatment of ASD. The proposed inhibitor demonstrated dual-target activity, inhibiting HDAC2-mediated deacetylation and H3R-mediated synaptic transmission irregularity. Experimental validation is warranted to develop it as an effective drug against ASD.

## 1 Introduction

Autism spectrum disorder (ASD) is a term used to encompass a collection of early-emerging deficits in repetitive sensory-motor behaviors and social communication. These traits have a strong genetic basis and may also have other causes [1]. While individuals with ASD can be highly diverse, this disorder is consistently characterized by the core features in two main areas: restricted, repetitive sensory-motor behaviors and social communication. These characteristics persist across different cultures, races, ethnicities, and socioeconomic groups [2]. ASD is attributed to alterations in early neural reorganization and brain development [3], 4]. Presently, autism is viewed as a spectrum ranging from mild to severe. However, many individuals with ASD, though not all, require ongoing support throughout their lives. ASD imposes a significant economic burden, primarily due to the need for continued assistance for adults who cannot function independently. This leads to increased healthcare and educational costs and results in a loss of income for caregivers [5]. There has been no *in vivo* exploration of the relationship between histone deacetylases (HDACs) and restricted and repetitive behavior patterns in humans. Preclinical studies, however, indicate that changes in HDAC levels during postnatal development by conditional knockout or HDAC overexpression can result in heightened repetitive behaviors in rodents [6]. This highlights the role of HDACs in ASD.

HDACs are members of the epigenetic regulator family and have long been considered promising therapeutic targets. HDACs can be categorized into five distinct classes: class I includes HDAC1, 2, 3, and 8; class IIa includes HDAC4, 5, 7, and 9. Class II includes HDAC6 and 10; class III includes sirtuins SIRT1–7; and class IV comprises HDAC11 [7]. Analysis of clinical trial trends has revealed the participation of multiple HDAC inhibitors [8]. Over the past 30 years, the scientific community has shown significant interest in HDAC modulation, leading to the development of 5 FDA-approved drugs that specifically target HDACs, with numerous new candidates currently under development [8], 9]. Among the 11 HDAC isoforms, histone deacetylases 2 (HDAC2) plays a crucial role in regulating excitatory amino acid transporter 2 (EAAT2) protein and mRNA levels, thereby governing synaptic plasticity and ameliorating cognitive and behavioral abnormalities [10]. The biochemical and structural characteristics of the HDAC2 active site can be summarized as follows: it forms a narrow, pit-like invagination at the center of multiple loops, with a Zn^2+^ ion located at the bottom of the pit. The catalytic pocket of HDAC2 is occupied by a Zn^2+^ that forms metal-coordinate bonds with Asp 181, His 183, and Asp 269. This pocket is connected to the surface pore via a tube-like hydrophobic invagination, which includes Gly154, Phe210, Phe155, and Leu276. Located adjacent to the catalytic site, a foot pocket (Met35, Arg39, Leu144, and Phe114) serves as an anchor for inhibitors or polar substrates [11].

Histamine is a neurotransmitter in the central nervous system (CNS) and modulates various physiological processes, including innate and acquired immunity, circulatory functions, hematopoiesis, and cell proliferation [12]. Recently, their interest has grown in studying histamine in the CNS and its impact on behavior under both normal physiological conditions and in the context of brain disorders [13], 14]. Consequently, the histaminergic system is considered a new pharmacological target for therapeutic interventions, leading to efforts to develop drugs that can act on different histamine receptors (H1R, H2R, H3R, and H4R) [15]. In an animal model of schizophrenia (SCH), an H3R antagonist has shown promise in alleviating behavioral impairments, including spatial working memory deficits. Similar abnormalities are observed in patients with ASD [16], 17]. H3R is a class A G-protein coupled receptor (GPCR) with a seven-transmembrane topology, featuring an extracellular N-terminus, an intracellular C-terminus, and connecting loops. Its transmembrane structure creates a conserved binding pocket where histamine binds via an ionic lock between its amine and Asp114 in TM3 [18]. Antagonists interact extensively within this pocket, involving residues like Asp114, polar residues in TM5 (Thr119, Asn121) and TM6 (Tyr374, Thr378), along with an aromatic cluster (Phe193, Phe396, Phe397) that engages in π-π stacking. A sodium-binding site involving Asp80 and Ser121 allosterically influences antagonist affinity, enabling high selectivity and inverse agonism. Recent studies show that H3R antagonism improves social behavior in rodents exposed to phencyclidine, with implications for ASD [19]. Ligands targeting H3R are also being considered as potential therapeutic agents for treating brain disorders, including schizophrenia (SCH), Alzheimer’s disease, and narcolepsy [20], [21], [22].

In this investigation, we explored the impact of phytocompounds derived from *Berberis vulgaris* on two targets associated with ASD. 26 compounds were employed to target histone deacetylase 2 (HDAC2) and the human histamine H3 receptor (H3R). We predicted the comprehensive toxicological profiles of these phytocompounds, along with their absorption, distribution, metabolism, and excretion (ADME) properties. Additionally, we forecasted their pharmacokinetics and drug-like properties. Virtual screening using molecular docking identified cinnamyl acetate (CA) as the lead compound. Molecular dynamics (MD) simulations of CA were conducted further to elucidate its dynamics and stability under physiological conditions.

## 2 Materials and methods

### 2.1 Preparation of a library of phytocompounds of *B. vulgaris*

A comprehensive exploration of the literature on *B. vulgaris* yielded a collection of 26 compounds [23], 24]. The SDF files for the phytocompounds were obtained from the PubChem database. Supplementary Table S1 provides detailed information about them. Utilizing Chimera 1.14, the SDF files were converted to PDB format. Subsequently, AutoDockTools-1.5.6 was employed to generate PDBQT files from the PDB files for molecular docking purposes [25].

### 2.2 Prediction of ADMET properties using pkCSM

To predict ADMET properties, Canonical SMILES of phytocompounds of *B. vulgaris* were retrieved from the PubChem database and then utilized as input for predicting ADMET properties through pkCSM [26]. Many ADMET parameters were extracted from pkCSM, and a subset of essential parameters was chosen for further analysis. Insights from ADMET properties are valuable for assessing the effectiveness and safety of compounds, particularly during the early stages of compound selection in drug discovery. This server facilitates the prediction of toxicity extent in both human and animal models. Understanding the potential adverse or toxic effects of drugs is crucial information in the early stages of drug design [27].

### 2.3 Prediction of pharmacokinetics and drug likeliness using SwissADME

The Canonical SMILES of phytocompounds of *B. vulgaris* obtained from PubChem were utilized as input for predictions using SwissADME [28]. We selected two sets of predictions from SwissADME: pharmacokinetics and drug-like properties. Various rules, including the Lipinski and Ghose rules, outline specific criteria that compounds must meet before progressing to successful drug development. These rules serve as essential benchmarks in evaluating the potential of compounds to become viable drugs.

### 2.4 Preparation of target proteins of autism spectrum disorder

In this research, key target proteins for autism spectrum disorder, histone deacetylase 2 (HDAC2) and the human histamine H3 receptor (H3R), were identified [11]. Our goal was to investigate the potential of phytocompounds from *B. vulgaris* in targeting ASD. The protein structures were obtained from the Protein Data Bank (HDAC2 ID: 5IX0 and H3R ID: 7F61). To prepare the protein structures for subsequent analysis, we cleaned the structures. This process involved removing all extraneous atoms, including water molecules, inhibitors, and ions, while preserving the protein’s structural integrity. The catalytic Zn^2+^ ion was also removed during virtual screening to identify compounds capable of occupying the active-site topology, consistent with the binding pose of the control inhibitor 6EZ. It’s noteworthy that the crystal structure of HDAC2 contained an inhibitor, 6EZ ((3-exo)-N-(4-amino-4′-fluoro[1,1′-biphenyl]-3-yl)-8-oxabicyclo[3.2.1]octane-3-carboxamide), which served as a control to validate our molecular docking methodology and to guide molecular simulations.

### 2.5 Virtual screening using molecular docking

Molecular docking was employed to conduct a virtual screening of phytocompounds of *B. vulgaris*. The docking analysis was carried out using AutoDock Vina [29]. The structures of HDAC2 and H3R were obtained from the Protein Data Bank. The flexibility in the structures of the phytocompounds was introduced to ensure optimal binding conformations. The molecular docking process underwent an initial validation step. We extracted the inhibitor (6EZ) coordinates from the crystal structures of HDAC2 and redocked them to assess whether they bind at the binding site. Remarkably, 6EZ occupied the same binding position in HDAC2 as it was present in the crystal structure, confirming the reliability of our molecular docking methodology. The same docking method was used to dock all phytocompounds from *B. vulgaris*. We cleaned the protein to prepare the receptor molecule by removing all atoms except those of the protein. Further receptor preparation was achieved by adding the polar hydrogens and Kollman charges utilizing AutoDockTools-1.5.6 [30]. We set a grid spacing of 1 Å. The center of the grid was *x* = 61.474, *y* = 25.985, and *z* = −2.295, and the grid size was *x* = 56 Å, *y* = 56 Å, and *z* = 58 Å for HDAC2. The center of the grid was *x* = −20.372, *y* = 51.12, and *z* = −0.682, and the grid size was *x* = 20 Å, *y* = 22 Å, and *z* = 20 Å for H3R. The exhaustiveness was set to its default value of 8. Top 9 binding poses were generated for each ligand, and the pose with the most favourable binding energy (lowest kcal/mol) was selected for further analysis. The docking analysis was conducted using PyMOL and Discovery Studio. The value of the binding constant (*K*_b_) was calculated using the eq.:

$$\Delta G=-RT\;\ln\left(K_{b}\right)$$

Where, Δ*G* is the binding energy; *R* is the universal gas constant (1.987 cal/mol/K); and *T* is the temperature (298.15 K). The value of the inhibition constant (*K*_i_) was calculated using the eq.:

$$\Delta G=RT\;\ln\left(K_{i}\right)$$

### 2.6 Molecular dynamics simulation

Cinnamyl acetate (CA) was selected based on criteria including toxicity, drug-likeness, pharmacokinetics, binding site, and binding energy. Molecular dynamics (MD) simulations were employed further to investigate the stability and dynamics of CA complexes. We also included the inhibitor of HDAC2 (6EZ) for molecular simulation studies that served as a control to compare the results of CA. The simulations were conducted using gromacs 2018.1 [31]. The topology of target proteins was generated using the pdb2gmx utility of gromacs-2018.1. For ligands or inhibitors, the topology was created utilizing the Antechamber packages of AmberTools21 [32]. The Amber99sb-ILDN force field was applied to simulate all systems [33]. The protonation states of all protein residues were assigned by the *gmx pdb2gmx* utility using the AMBER99SB-ILDN force field at a pH of 7.0. Ligand topologies, including protonation states and partial charges, were generated using the Antechamber package from AmberTools21 with the AM1-BCC charge method, also at pH 7.0. Manual merging of ligand and protein topologies was performed to generate the topology of the complexes. Triclinic boxes were created around the structures with a 10 Å distance between the structure and the box edge to satisfy periodic boundary conditions (PBC). The systems were solvated with water using the TIP3P water model. The system charges were then neutralized by adding an equal amount of counterions (Na^+^ or Cl^−^). Additionally, 150 mM NaCl was introduced to mimic physiological salt concentration. The system’s energy was minimized to eliminate weak van der Waals forces. Two sets of equilibrations were performed before simulating all systems. The first equilibration was performed using the NVT ensemble (constant volume, temperature, and number of particles) at 310 K with a V-rescale thermostat for 1 ns [34]. The second equilibration utilized the NPT ensemble (constant number of particles, pressure, and temperature) at 1.0 bar with a Parrinello-Rahman barostat for the next 1 ns [35]. The equilibrated systems were then used in a 100 ns simulation at 310 K, and PBC corrections were applied to all trajectories before analysis. Control simulations were performed for apoproteins. Various studies were conducted using gromacs utilities, and the components of binding energy were computed using the g_mmpbsa package [36].

## 3 Results

### 3.1 Toxicity of phytocompounds of *B. vulgaris*

Toxicity predictions for all compounds of *B. vulgaris* were performed using pkCSM, considering parameters such as oral rat acute toxicity, Ames toxicity, hepatotoxicity, skin sensitization, hERG I inhibition, minnow toxicity, and *T. Pyriformis* toxicity. Data interpretation adhered to the server’s guidelines, and the predicted toxicological properties are summarized in Table 1. Among the compounds, only three (berberine, palmatine, and 2-methoxy-4-vinylphenol) exhibited AMES toxicity. None of the phytocompounds were predicted to be hERG I inhibitors, while three were found to be forecasted as hERG II inhibitors. The oral rat acute toxicity of all compounds was below the permissible limits. Only one compound, 3-ethoxypropionaldehyde, was predicted to be toxic in the *T. Pyriformis* toxicity model. In the Minnow toxicity model, approximately 34 % of compounds (9 of 26) were toxic. The pkCSM provided LC_50_ values that predict lethality for Flathead Minnows, using a model trained on LC50 measurements for 554 compounds. CA has been documented to restore the methamphetamine-induced toxicity in albino rats, in which alanine aminotransferase, aspartate aminotransferase, alkaline phosphatase, cholesterol, bilirubin, triglycerides, and low-density lipoprotein were measured [37]. Moreover, several *in vitro* and *in vivo* studies have found the shielding effect of CA against toxicities induced by chemicals, including microbial toxins, metals, pharmaceuticals, and pesticides, by protecting the cell’s survival through the reduction of nitric oxide overproduction and reactive oxygen species, as well as by suppression of pro-apoptotic signaling [38]. Overall, CA is well known to protect against numerous toxicities and hence can be developed as a safe and effective drug.

**Table 1:** Toxicity assessment of the compounds of *Berberis vulgaris* using the pkCSM webserver.

S. no.	Name of phytocompounds	AMES tox.	hERG I inhibitor	hERG II inhibitor	ORAT (LD50)	*T. Pyriformis* toxicity	MT (log mM)
1	Cinnamyl acetate	No	No	No	337.45	1.03	0.11
2	*trans*-Sesquisabinene hydrate	No	No	No	369.80	1.77	0.46
3	Ylangene	No	No	No	335.96	1.12	0.13
4	Sesquicineole	No	No	No	394.71	1.19	0.60
5	2-Hydroxycinnamic acid	No	No	No	359.84	0.32	1.61
6	Alpha-longipinene	No	No	No	342.71	0.91	0.56
7	Guaiol	No	No	No	397.82	1.25	0.91
8	Alpha-curcumene	No	No	No	378.98	1.87	−0.62
9	Alpha-guaiene	No	No	No	343.12	1.48	0.37
10	Guaia-1-10-11-diene	No	No	No	315.12	1.62	0.44
11	Berberine	Yes	No	No	864.80	0.35	−0.28
12	Cinnamyl alcohol	No	No	No	257.62	−0.13	1.87
13	Columbamine	No	No	Yes	815.84	0.40	0.08
14	7-10-Pentadecadiynoic acid	No	No	No	345.42	0.93	−0.10
15	Stigmasterol	No	No	Yes	1,048.26	0.43	−1.68
16	Methyl-5-7-hexadecadiynoate	No	No	No	367.35	2.14	−0.68
17	Oxyberberine	No	No	No	841.15	0.41	0.17
18	Lambertine	No	No	No	777.31	0.81	0.45
19	Mesitylene	No	No	No	219.60	0.25	1.21
20	Palmatine	Yes	No	Yes	899.00	0.39	−0.31
21	Phenylethyl alcohol	No	No	No	229.80	−0.39	2.05
22	Guaiacol	No	No	No	262.80	−0.13	1.95
23	2-Methoxy-4-vinylphenol	Yes	No	No	311.77	0.07	1.96
24	Stigmasterolglucoside	No	No	No	1,478.50	0.29	−0.68
25	2-Pentenoic acid	No	No	No	164.59	−0.47	2.02
26	3-Ethoxypropionaldehyde	No	No	No	196.20	−0.76	2.30

AMES tox. is AMES toxicity, ORAT is oral rat acute toxicity in g/kg, MT is minnow toxicity, *T. Pyriformis* toxicity is in log µg/L.

### 3.2 ADME properties of phytocompounds of *B. vulgaris*

The ADME properties of the phytocompounds of *B. vulgaris* were assessed using pkCSM, considering two parameters for absorption (water solubility and intestinal absorption), two for distribution (fraction unbound and VDss, i.e., volume of distribution), two parameters for metabolism (CYP3A4 substrate and CYP3A4 inhibitor), and two excretion parameters (renal OCT2 substrate and total clearance). The predicted ADME of the properties of compounds of *B. vulgaris* are detailed in Supplementary Table S2. Notably, no compound displayed low intestinal absorption. Seven compounds, including CA, exhibited good water solubility, two compounds (namely 2-hydroxycinnamic acid and mesitylene) exhibited moderate water solubility, and the remaining were insoluble in water. The distribution parameters (VDss and fraction unbound) of CA were moderate. 12 out of 26 compounds were found as CYP3A4 substrates, while the remaining (including CA) were not predicted as CYP3A4 substrates. Likewise, 12 compounds were predicted to be CYP3A4 inhibitors. Only two compounds (oxyberberine and lambertine) were identified as substrates of Renal OCT2.

### 3.3 Pharmacokinetics and drug-like properties of phytocompound of *B. vulgaris*

The pharmacokinetics and drug-like properties of *compounds from B. vulgaris* were predicted using SwissADME. The drug-like properties are listed in Supplementary Table S3. Lipinski’s Rule of Five, established by Christopher A. Lipinski in 1997, provides a guideline for assessing the drug-likeness of compounds intended for oral administration in humans. This rule suggests that such a compound should generally be moderately lipophilic and relatively small [39]. Notably, 17 compounds (including CA) did not violate the Lipinski Rule of Five, whereas 9 phytocompounds from *B. vulgaris* violated only one rule. The Ghose filter, another knowledge-based filter used in designing chemistry libraries for drug discovery, imposes stricter criteria: molecular weight between 160 and 480 Da, number of atoms between 20 and 70, logP between −0.4 and 5.6, and molar refractivity between 40 and 130 [40]. Three compounds showed one Ghose filter violation, while 6 compounds showed three or more violations of the Ghose filter. The remaining compounds (including CA) did not violate any of the Ghose filter parameters.

The pharmacokinetics of compounds *from B. vulgaris* were assessed using SwissADME, focusing on three key parameters: gastrointestinal absorption, blood-brain barrier permeability, and P-glycoprotein substrate status. The pharmacokinetic properties of the phytocompounds are listed in Supplementary Table S3. A total of 7 compounds exhibited low gastrointestinal absorption, and the remaining (including CA) exhibited high gastrointestinal absorptivity. Of the 26 compounds, only 6 were not predicted to be blood-brain barrier permeants. It should be kept in mind that a compound should be able to penetrate the blood-brain barrier to be a successful candidate against neurological disorders like autism spectrum disorder (ASD). CA was predicted to be blood-brain barrier-permeant. Nearly 23 % of phytocompounds (6 out of 26) were found as the substrate of P-glycoprotein. In summary, CA, the identified lead compound, exhibited favorable drug-like properties and pharmacokinetics, suggesting its potential for successful drug development for ASD. Furthermore, numerous *in vitro* and *in vivo* studies have demonstrated CA’s protective effects against toxicities induced by various substances. This protection is achieved by many mechanisms, including safeguarding cell survival through the reduction of nitric oxide and reactive oxygen species overproduction [38]. In summary, CA is widely acknowledged for its ability to shield against a variety of toxicities, suggesting its potential development as a safe and effective drug.

### 3.4 Virtual screening through molecular docking

Initially, the molecular docking method was validated by retrieving the inhibitor (6EZ) from the bound crystal structure of HDAC2. The inhibitor was found at the same binding position as observed in the crystal structure when docked (RMSD = 0.135 Å). The validation of H3R was also done with the co-crystal antagonist (PF-03654746). The root-mean square deviation (RMSD) was 0.067 Å. It is important to note that the molecular docking of HDAC2 was performed without the catalytic Zn^2+^ ion. This approach successfully identified potential compounds that stably occupy the active site cavity. It does not show the interactions with the metal center (Zn^2+^), a key feature for many potent HDAC inhibitors. Therefore, the binding affinity calculated here should be interpreted as a measure of favorable cavity occupancy and interaction with the surrounding residues of the protein. Future work involving docking with Zn^2+^-bound HDAC2 is required to predict potential inhibitory activity more accurately.

For clarity, the active site of HDAC2, its metal coordination, and the ligand-binding regions of H3R are presented in Supplementary Figure S1. Molecular docking analysis of phytocompounds from *B. vulgaris* was conducted against two distinct proteins (HDAC2 and H3R) implicated in autism spectrum disorder (ASD). The corresponding values for binding energies and binding sites are listed in Table 2. The binding energy of cinnamyl acetate (CA) was highest towards HDAC2 among compounds of *B. vulgaris*. In the H3R docking assay, *trans*-sesquisabinene hydrate exhibited the highest binding affinity. To identify the lead compound, we considered the average binding energy of the two proteins (HDAC2 and H3R). Based on average binding energy, cinnamyl acetate (CA) emerged as the most potent phytocompounds from *B. vulgaris* against the selected ASD target proteins. Hence, in this section, we have discussed only the binding of CA to HDAC2 and H3R.

**Table 2:** Results obtained from the molecular docking of compounds of *Berberis vulgaris* using AutoDock Vina.

Name of phytocompounds	HDAC2				H3R				Av BE
	BE	*K*_b_	*K*_i_	BS	BE	*K*_b_	*K*_i_	BS	
Cinnamyl acetate	−7.4	26.76	3.74	AS	−7.6	37.52	2.67	AS	−7.5
*trans*-Sesquisabinene hydrate	−5.7	1.52	65.97	AS	−9	399.07	0.25	AS	−7.35
Ylangene	−6.7	8.21	12.19	AS	−7.8	52.59	1.90	AS	−7.25
Sesquicineole	−5.6	1.28	78.11	AS	−8.3	122.36	0.82	AS	−6.95
2-Hydroxycinnamic acid	−6.6	6.93	14.43	AS	−7.2	19.09	5.24	AS	−6.9
Alpha-longipinene	−6.5	5.85	17.08	AS	−7.3	22.60	4.42	AS	−6.9
Guaiol	−7.1	16.12	6.20	AS	−6.7	8.21	12.19	AS	−6.9
Alpha-curcumene	−6.7	8.21	12.19	AS	−7	13.62	7.34	AS	−6.85
Alpha-guaiene	−6.7	8.21	12.19	AS	−7	13.62	7.34	AS	−6.85
Guaia-1-10-11-diene	−6.6	6.93	14.43	AS	−6.8	9.72	10.29	AS	−6.7
Berberine	−7.3	22.60	4.42	AS	−5.9	2.12	47.06	AS	−6.6
Cinnamyl alcohol	−6.5	5.85	17.08	AS	−6.5	5.85	17.08	AS	−6.5
Columbamine	−6.9	11.50	8.69	OS	−6.1	2.98	33.57	AS	−6.5
7-10-Pentadecadiynoic acid	−5.2	0.65	153.48	AS	−7.7	44.42	2.25	AS	−6.45
Stigmasterol	−7	13.62	7.34	OS	−5.6	1.28	78.11	AS	−6.3
Methyl-5-7-hexadecadiynoate	−4.8	0.33	301.61	AS	−7.5	31.69	3.16	AS	−6.15
Oxyberberine	−7.2	19.09	5.24	OS	−5.1	0.55	181.72	AS	−6.15
Lambertine	−7.2	19.09	5.24	AS	−4.8	0.33	301.61	AS	−6
Mesitylene	−5.1	0.55	181.72	AS	−6.7	8.21	12.19	AS	−5.9
Palmatine	−7	13.62	7.34	OS	−4.5	0.20	500.58	AS	−5.75
Phenylethyl alcohol	−5.3	0.77	129.63	AS	−6	2.52	39.75	AS	−5.65
Guaiacol	−5.7	1.52	65.97	AS	−5.5	1.08	92.48	AS	−5.6
2-Methoxy-4-vinylphenol	−5	0.46	215.16	AS	−6.1	2.98	33.57	AS	−5.55
Stigmasterolglucoside	−7.4	26.76	3.74	OS	−3.6	0.04	2,288.6	AS	−5.5
2-Pentenoic acid	−5	0.46	215.16	AS	−5	0.46	215.16	AS	−5
3-Ethoxypropionaldehyde	−4.1	0.10	983.69	AS	−4	0.09	1,164.6	AS	−4.05

HDAC2 is histone deacetylase 2, H3R is human histamine H3 receptor, BE is binding energy in kcal/mol, *K*_b_ is binding constant in × 10^4^ M^−1^, *K*_i_ is inhibition constant in µM, AS is active site, BS is binding site, OS is site other than binding site, and Av BE is average binding energy.

**Figure 1:**
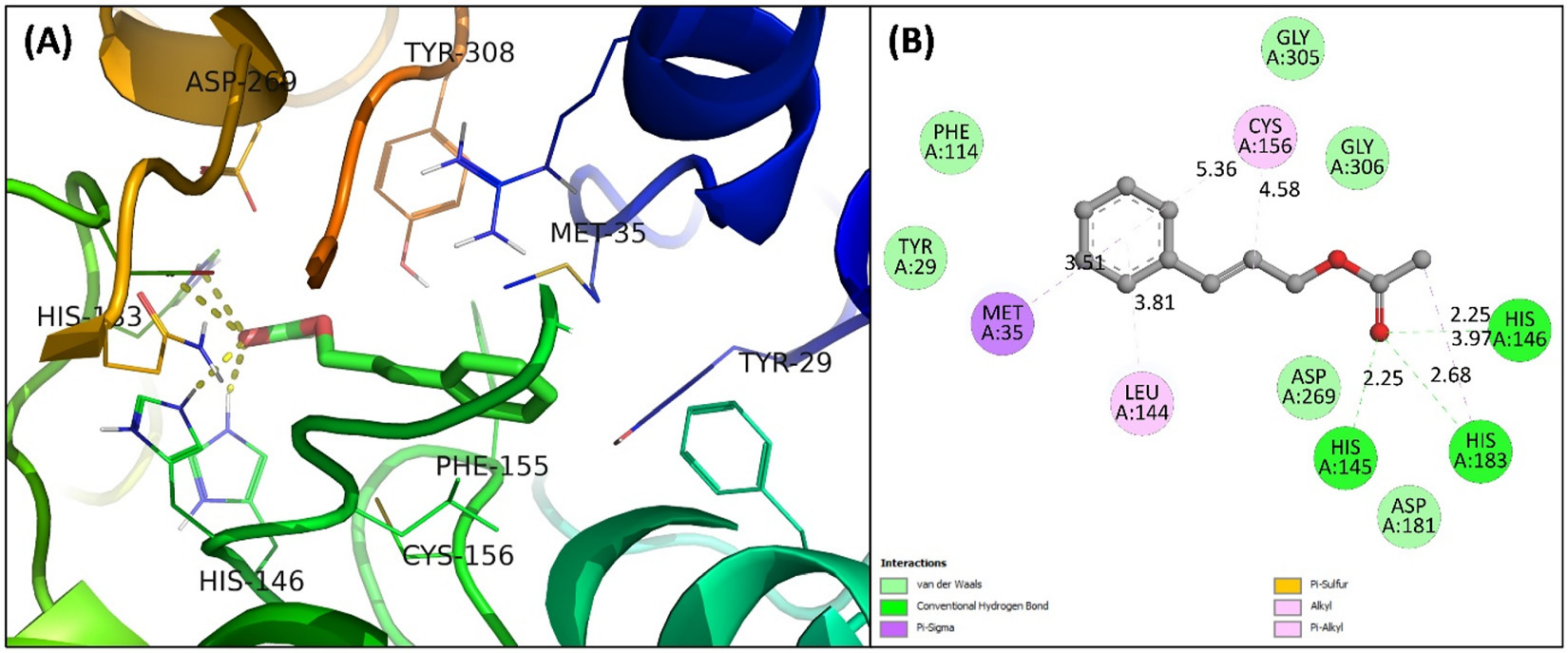
Docked pose of the HDAC2-CA complex. (A) Three-dimensional representation of the HDAC2-CA complex. CA is shown as sticks. HDAC2 is shown as a coloured ribbon, with interacting residues highlighted. (B) Two-dimensional representation of the HDAC2-CA complex prepared using Discovery Studio 2021.

The docking of CA with HDAC2 resulted in a binding energy of −7.4 kcal/mol, corresponding to a binding constant (*K*_b_) of 2.67 × 10^5^ M^−1^. CA was precisely docked into the active site of HDAC2, overlapping with the protein’s 6EZ inhibitor. Three hydrogen bonds were formed by His145, His146, and His183 of HDAC2, with a CA bond length of 2.25, 2.25, and 2.68 Å, respectively (Figure 1). The van der Waals forces also stabilized the complex involving Tyr29, Phe114, Gly305, and Gly306. Moreover, hydrophobic forces (Met35, Leu144, Cys156) also played a remarkable role in the interaction. Histone deacetylase (HDAC) enzymes, members of the epigenetic regulatory group, have long been identified as promising therapeutic targets. Among the 11 HDAC isoforms, HDAC2 plays a crucial role in regulating the mRNA and protein levels of EAAT2. Over the past three decades, there has been substantial interest within the scientific community in modulating HDACs. This has led to the development of 5 drugs approved by the FDA explicitly targeting HDACs, with an increasing number of new candidates under scrutiny [8], 9]. The biochemical and structural characteristics of the active site of HDAC2 can be summarized as follows: it forms a narrow, pit-like invagination at the center of multiple loops, with a Zn^2+^ ion located at the bottom of the pit. The catalytic pocket of HDAC2 is occupied by a Zn^2+^ that forms metal-coordinate bonds with Asp 181, His 183, and Asp 269. This pocket is connected to the surface pore via a tube-like hydrophobic invagination, which includes Phe155, Gly154, Leu276, and Phe210. Located right next to the catalytic site, there is a foot pocket (Met35, Arg39, Leu144, and Phe114) that serves as an anchor for inhibitors or polar substrates [11]. The interaction of CA at the active site and with catalytic-pocket residues could lead to inhibition of HDAC2. This inhibition could be employed to manage autism spectrum disorder.

**Figure 2:**
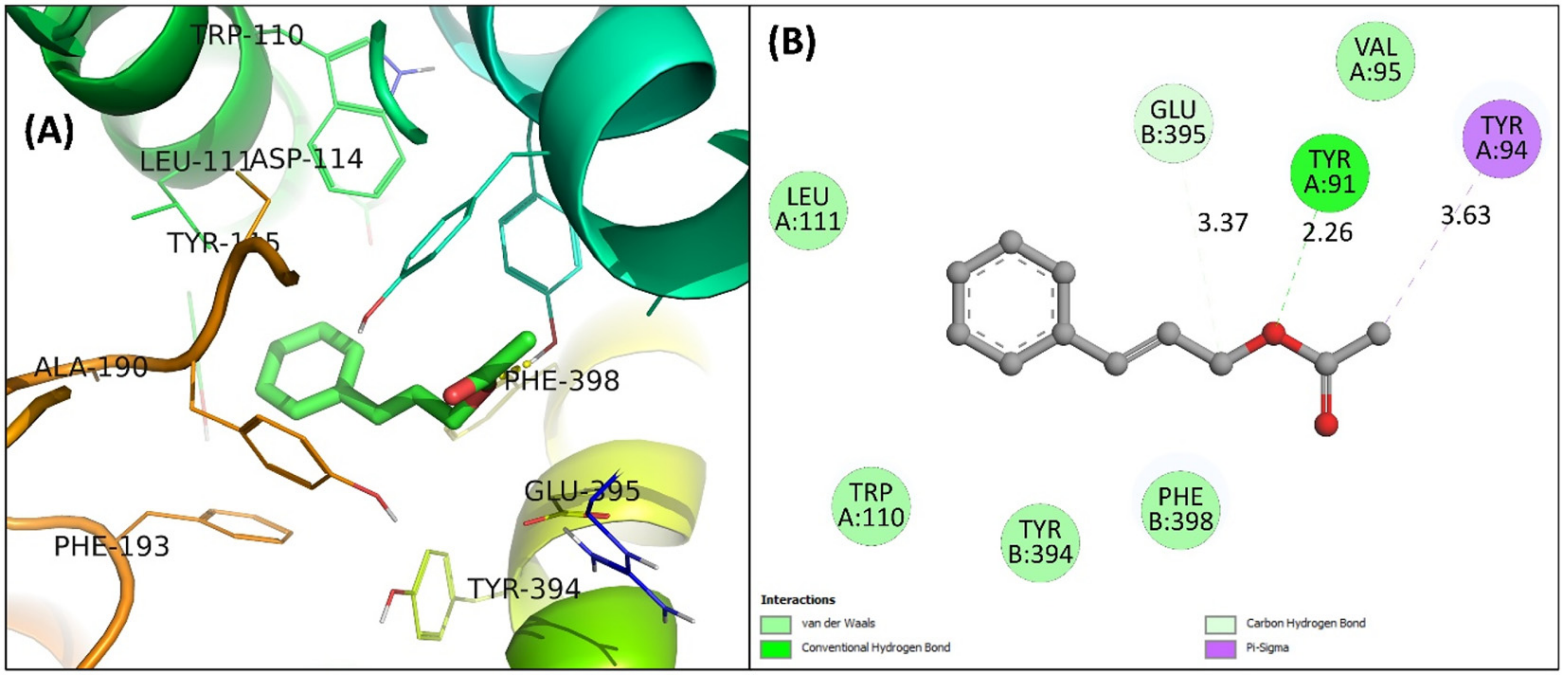
Docked pose of the H3R-CA complex. (A) Three-dimensional representation of the H3R-CA complex. CA is shown as sticks. H3R is shown as a coloured ribbon, with interacting residues highlighted. (B) Two-dimensional representation of the H3R-CA complex prepared using Discovery Studio 2021.

The histamine H3 receptor (H3R), a member of the G protein-coupled receptors superfamily, was first recognized as a presynaptic autoreceptor responsible for regulating histamine release and synthesis in the brain [41]. H3R is an essential target in disorders associated with neuron function and degeneration [42]. Hence, we also included the H3R to target ASD. The binding energy for the complexation of CA with H3R was −7.6 kcal/mol, corresponding to a binding constant (*K*_b_) of 3.75 × 10^5^ M^−1^. Tyr91 and Glu395 of H3R formed hydrogen bonds with CA with bond lengths of 2.26 and 3.37 Å, respectively (Figure 2). One hydrophobic interaction with Tyr94 was formed during complexation. The H3R-CA complex was also stabilized by van der Waals interactions involving Trp110, Leu11, Tyr394, and Phe398. Asp114, Trp110, Trp291, and Phe192 primarily characterize the histamine binding site. This binding site is also shared by H3R antagonists such as thioperamide, ciproxifan, ABT239, and pitolisant [43], 44]. The interaction of CA at the active site of H3R could block downstream signaling and ultimately ameliorate autistic-like symptoms [45].

### 3.5 Molecular simulations

#### 3.5.1 Examinations of fluctuations and deviations in HDAC2 and H3R

**Figure 3:**
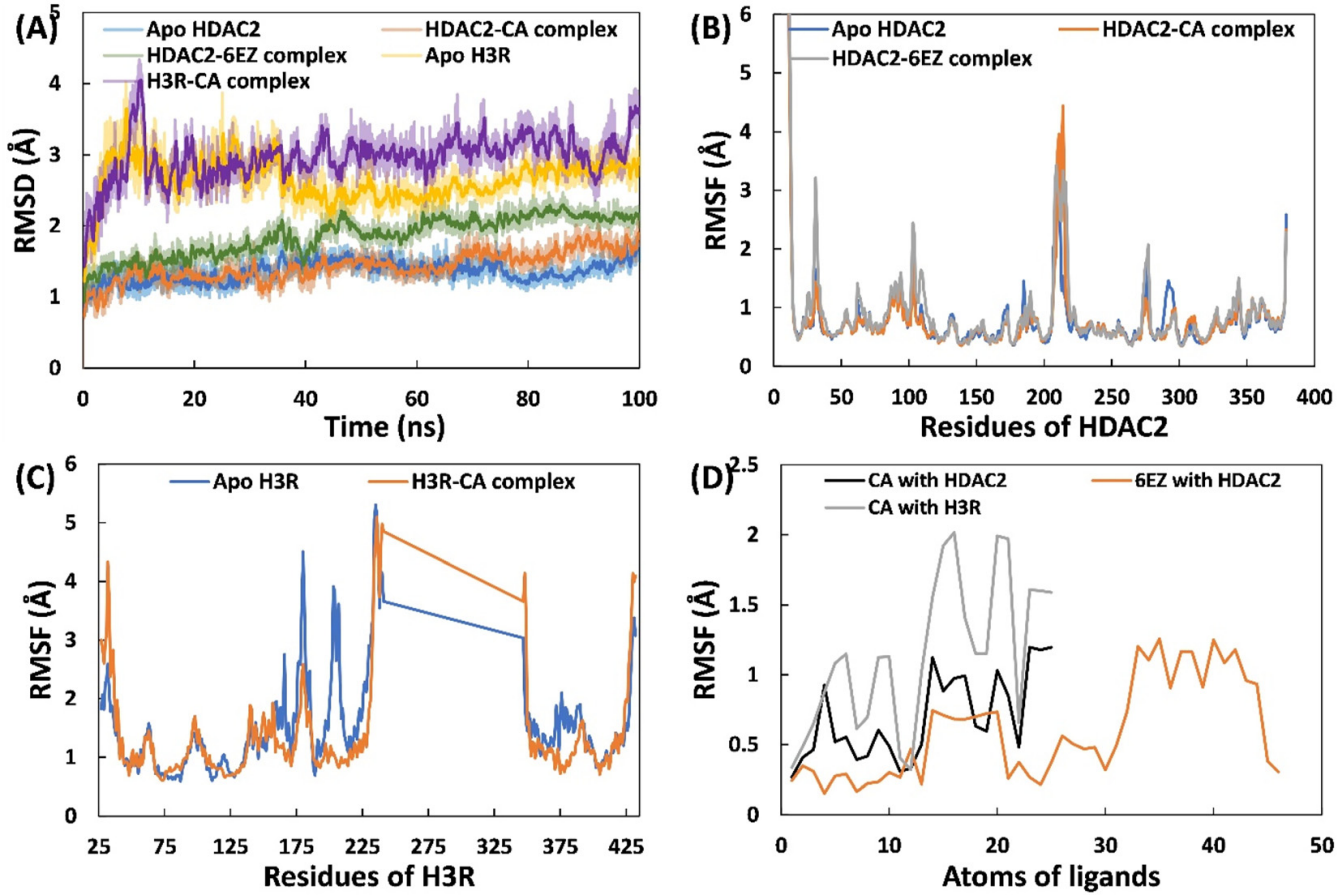
Analysis of fluctuations and deviations in HDAC2/H3RCA upon binding of CA. (A) RMSD of backbone atoms of apo HDAC2, HDAC2-CA complex, HDAC2-6EZ complex, apo H3R, and H3R-CA complex over simulation time. (B) RMSF of C_α_ atoms of HDAC2 in the absence and presence of CA or 6EZ. (C) RMSF of C_α_ atoms of H3R in the absence and presence of CA. (D) RMSF of individual atoms of Ca and 6EZ in complex with HDAC2 and H3R.

MD simulations were conducted to explore CA’s dynamics and stability in the presence of the target proteins. The primary analysis involved calculating the root-mean-square deviation (RMSD) of each trajectory relative to the initial structure, and the results are shown in Figure 3A. For apo HDAC2, the RMSD remained stable throughout the simulation. The findings show the protein reached equilibrium during the equilibration phases. A similar result was obtained for the HDAC2-CA complex. However, the HDAC2-6EZ complex exhibited greater variability and reached stability at approximately 50 ns. The average RMSD for apo HDAC2, HDAC2-CA complex, and HDAC2-6EZ complex was 1.35 ± 0.15, 1.42 ± 0.21, and 1.85 ± 0.28 Å, respectively. For the Apo H3R and H3R-CA complexes, the system required nearly 20 ns to equilibrate, after which it became stable. The average RMSD for apo H3R and H3R-CA complex was 2.64 ± 0.30 and 2.96 ± 0.33 Å, respectively. These data provide preliminary evidence of the stability of the complexes in a physiological environment. A published report also supports the findings, where the RMSD of apo (unbound HDAC2) and pitolisant-bound HDAC2 remained below 2.75 Å [11].

The fluctuations in the residues of HDAC2 and H3R in both the uncomplexed and complexed states were also calculated. The root mean square fluctuation (RMSF) of nearly all residues of HDAC2 and its complexes with CA and 6EZ remained below 1 Å (Figure 3B). For example, the average RMSF values for apo HDAC2, the HDAC2-CA complex, and the HDAC2-6EZ complex were 0.763, 0.767, and 0.867 Å, respectively. A study revealed notable fluctuations in the variable loop regions of HDAC2, which constitute the orifice of the metal-dependent substrate-binding site, when the protein was simulated in the free form [11]. H3R showed relatively higher RMSF than HDAC2 (Figure 3C). The average RMSF for apo H3R and H3R-CA complex was 1.502 and 1.373 Å, respectively. The terminal segments of both proteins exhibited greater fluctuations because they are located at the ends, where they undergo greater conformational motion in an aqueous environment. The data indicate that most residues remained stable under physiological conditions, including in CA. The RMSF of individual atoms of CA and 6EZ was also calculated to examine their dynamics, as shown in Figure 3D. Both ligands exhibited fluctuations upon complexation with the target proteins. This fluctuation is attributed to the movement of ligands at the binding or active site of target proteins [46]. The overall RMSF of the ligand (CA) was less than 0.5 Å, indicating a stable binding of the ligand [11]. Notably, the CA-H3R interaction decreased the RMSF, providing further insight into the stability of the complex.

#### 3.5.2 Analysis of protein’s compactness and energies

**Figure 4:**
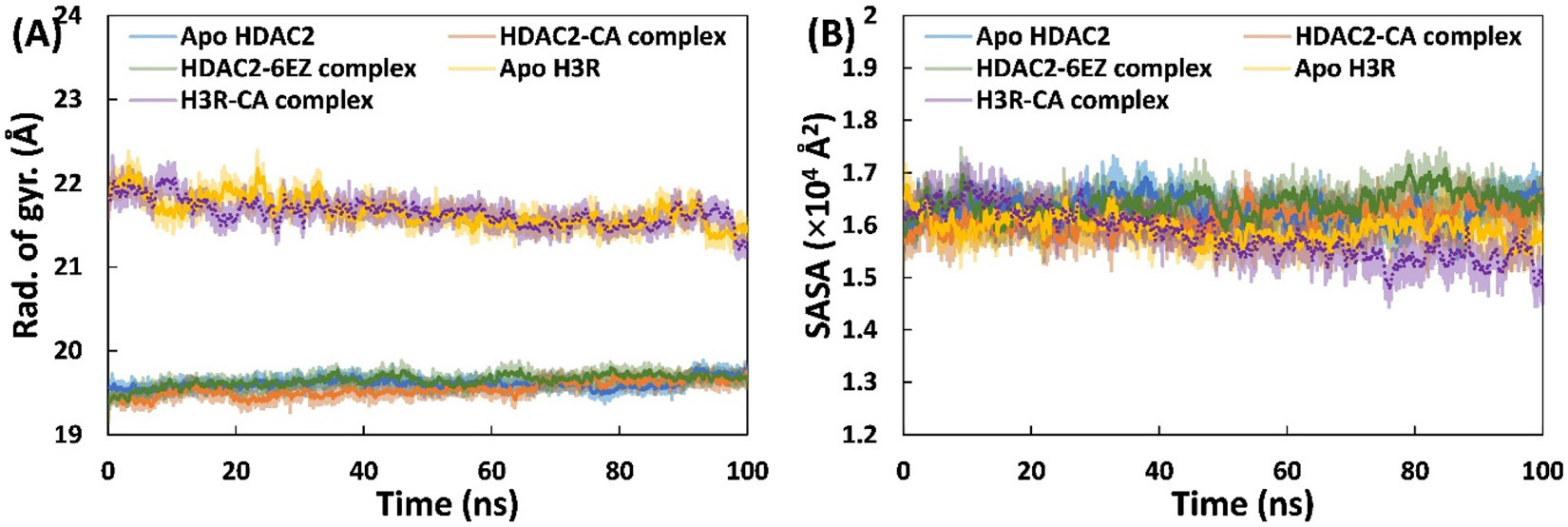
Effect of CA binding on compactness of HDAC2/H3R. (A) Radius of gyration of backbone atoms of apo HDAC2, HDAC2-CA complex, HDAC2-6EZ complex, apo H3R, and H3R-CA complex over simulation time. (B) Solvent accessible surface area of apo HDAC2, HDAC2-CA complex, HDAC2-6EZ complex, apo H3R, and H3R-CA complex over simulation time.

The stability of both proteins and their complexes was further assessed through the calculation of various parameters, including radius of gyration (*R*_g_), solvent-accessible surface area (SASA), and the energies (potential and total energies). The radius of gyration, a key metric, is defined as the root-mean-square (RMS) distance, considering mass, between a set of atoms and their shared center of mass. Typically, compact proteins display minimal fluctuations in *R*_g_ during simulations, while open or expanded proteins exhibit more significant changes in *R*_g_ values throughout the simulation [47]. These characteristic underscores the significance of *R*_g_ as a crucial parameter for evaluating the stability and structural compactness of proteins in molecular simulations [48]. Figure 4A illustrates the *R*_g_ of proteins and complexes over time. Notably, the *R*_g_ of HDAC2 and its complexes increased slightly over time and then stabilized. The average *R*_g_ values for apo HDAC2, the HDAC2-CA complex, and the HDAC2-6EZ complex were 19.60, 19.54, and 19.64 Å, respectively. However, *R*_g_ increased slightly over time for H3R and its complex. The average *R*_g_ for Apo H3R and H3R-CA complex is 21.66 and 21.65 Å, respectively. Although the *R*_g_ of H3R and its complex varied over time, they remained within an acceptable range. In summary, the calculation of *R*_g_ provides additional validation of the complexes’ stability. A study on the simulation of many ligands with HDAC2 found that the gyration radius remained steady over the simulation time [11].

SASA for all systems is shown in Figure 4B. SASA values stayed consistent across all systems during simulations under physiological conditions. The SASA trajectories of proteins and complexes remained stable throughout. For apo HDAC2, HDAC2-CA complex, and HDAC2-6EZ complex, the average SASA values were 1.62 × 10^4^, 1.61 × 10^4^, and 1.64 × 10^4^ Å^2^, respectively. Likewise, the average SASA for the apo H3R and H3R-CA complex were 1.59 × 10^4^ and 1.58 × 10^4^ Å^2^, respectively. The two proteins (HDAC2 and H3R) showed minimal differences in SASA compared to their respective CA complexes. The SASA analysis confirms the stability of CA within the complex structures containing the target proteins. Additionally, the total and potential energies of the systems were calculated (Supplementary Figure S2), and both remained steady throughout the simulation. The combined analysis of *R*_g_, SASA, and energies supports that the CA formed stable complexes with the test proteins (HDAC2 and H3R) under physiological conditions.

#### 3.5.3 Effect of CA on the secondary structure of target proteins

**Figure 5:**
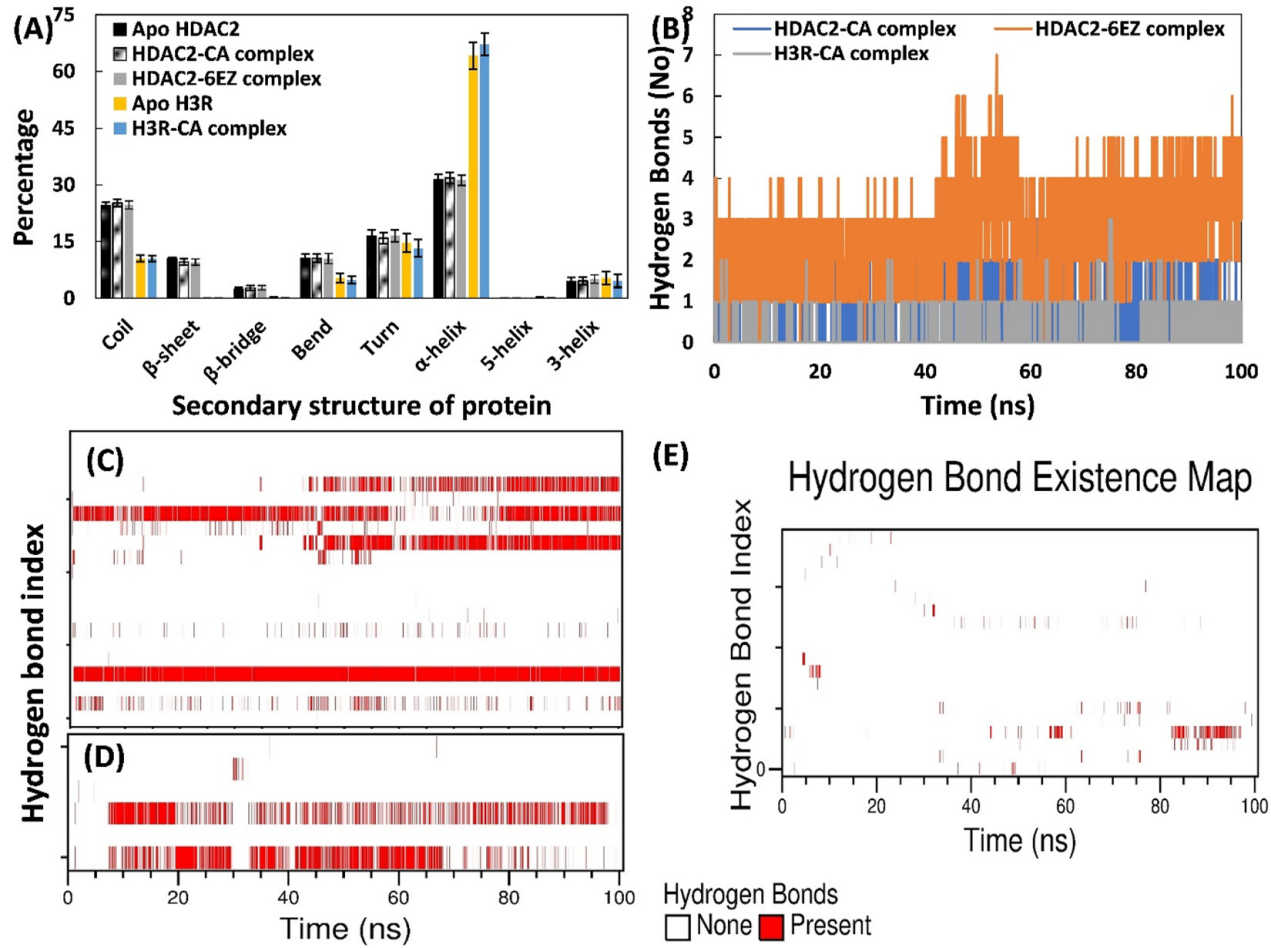
Effect of CA binding on the secondary structure of HDAC2/H3R. (A) Average secondary structure in HDAC2 and H3R in the absence and presence of CA or 6EZ. (B) Number of hydrogen bonds formed over simulation time in HDAC2-CA complex, HDAC2-6EZ complex, and H3R-CA complex. (C) Hydrogen bond existence map of HDAC2-CA complex. (D) Hydrogen bond existence map of HDAC2-6EZ complex. (E) Hydrogen bond existence map of H3R-CA complex.

The impact of CA binding on the structural stability of HDAC2 and H3R was evaluated by analyzing their secondary structures (Figure 5A). In apo HDAC2, the typical percentages of bends, coils, β-sheets, β-bridges, turns, α-helices, and 3′-helices were 10.49, 24.55, 10.45, 2.44, 16.33, 31.34, and 4.36 %, respectively. Interestingly, these secondary structural elements in the HDAC2-CA complex remained largely unchanged, with averages of 10.51, 25.17, 9.50, 2.69, 15.81, 31.80, and 4.48 %. Similarly, CA complexation had little effect on the secondary structure of H3R. The average percentages of coils, bends, turns, α-helices, and 3′-helices were 10.43, 5.31, 14.65, 64.14, and 5.25 %, respectively. In the H3R-CA complex, the secondary structures were coils at 10.41 %, bends at 4.718 %, turns at 13.15 %, α-helices at 67.15 %, and 3′-helices at 4.54 %. Overall, minimal changes were observed in the structures of HDAC2 and H3R upon CA binding. The analysis confirms that CA binding does not affect the stability of these target proteins.

#### 3.5.4 Analysis of hydrogen bonding between CA and target proteins

The interaction of CA with HDAC2 and H3R was examined by quantifying hydrogen-bond counts and analyzing the hydrogen-bond profile. Figure 5B illustrates the number of hydrogen bonds made by CA with HDAC2 and H3R. The average number of hydrogen bonds formed by CA with HDAC2 was 0.862. It is worth mentioning that the average number of hydrogen bonds formed by 6EZ (inhibitor) with HDAC2 was higher (2.68). The average number of hydrogen bonds made by CA H3R was very low, i.e., 0.19. The presence of hydrogen bonds was also monitored throughout each trajectory of the complexes. The data reveal consistency in hydrogen bond formation between CA and HDAC2 with some skips in a few frames (Figure 5C). The HDAC2-6EZ complex has a continuous presence of hydrogen bonds throughout the trajectory (Figure 5D). However, hydrogen bonds occurred only in a few frames of the H3R-CA complex (Figure 5E). Hydrogen bond occupancy for the complexes was also calculated. Arg39 exhibited hydrogen-bonding interactions with CA among all residues of HDAC2. Similarly, for the HDAC2-6EZ complex, Arg39 exhibited 97.1 % hydrogen bond occupancy, which was the highest among all pairs. This data confirms the involvement of hydrogen bonds in the complexation of CA with HDAC2 and H3R.

#### 3.5.5 Principal component analysis

**Figure 6:**
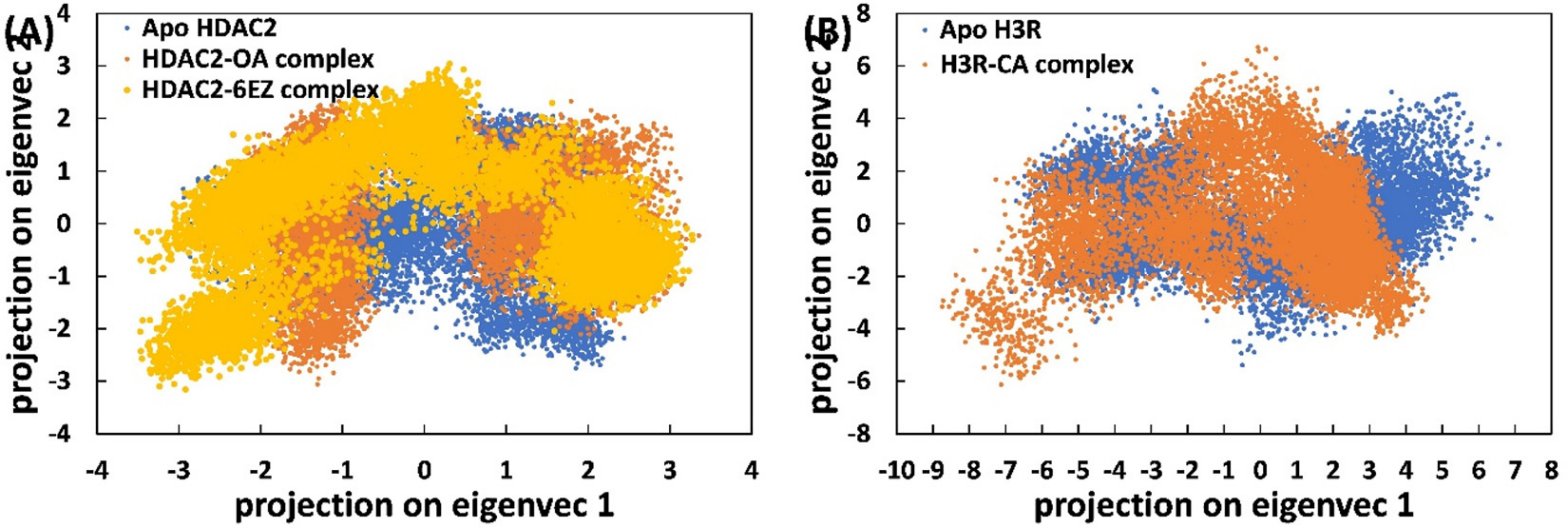
Effect of CA binding on the principal component analysis of HDAC2/H3R. (A) Two-dimensional projection of eigenvectors for principal component analysis of apo HDAC2, HDAC2-CA complex, and HDAC2-6EZ complex. (B) Two-dimensional projection of eigenvectors for principal component analysis of apo H3R and H3R-CA complex.

Principal component analysis (PCA) is a conventional statistical technique used to analyze large-scale protein movements. By reducing the dimensionality of data sets and retaining critical information in the form of eigenvectors, PCA allows for the examination of protein flexibility [49]. The dispersion of conformational spaces distinctly delineates the frequency of various conformations exhibited by a protein. The projection of eigenvectors for complexed and uncomplexed proteins in a two-dimensional space is presented in Figure 6. In the case of HDAC2, both the complexes (HDAC2-CA complex and HDAC2-6EZ complex) appeared to occupy a similar conformational space in the 2D projection of eigenvectors compared to ap HDAC2. This observation suggests that the flexibility of HDAC2 remained relatively similar after binding to CA or 6EZ; however, for H3R, the conformational space occupied by the complex was slightly larger. The elevated count of eigenvectors or the expanded conformational spaces characterizes the limited movement of H3R when it is bound to CA [11]. Overall, the data indicate that CA binding did not alter the flexibility of HDAC2 but slightly reduced the flexibility of H3R.

**Figure 7:**
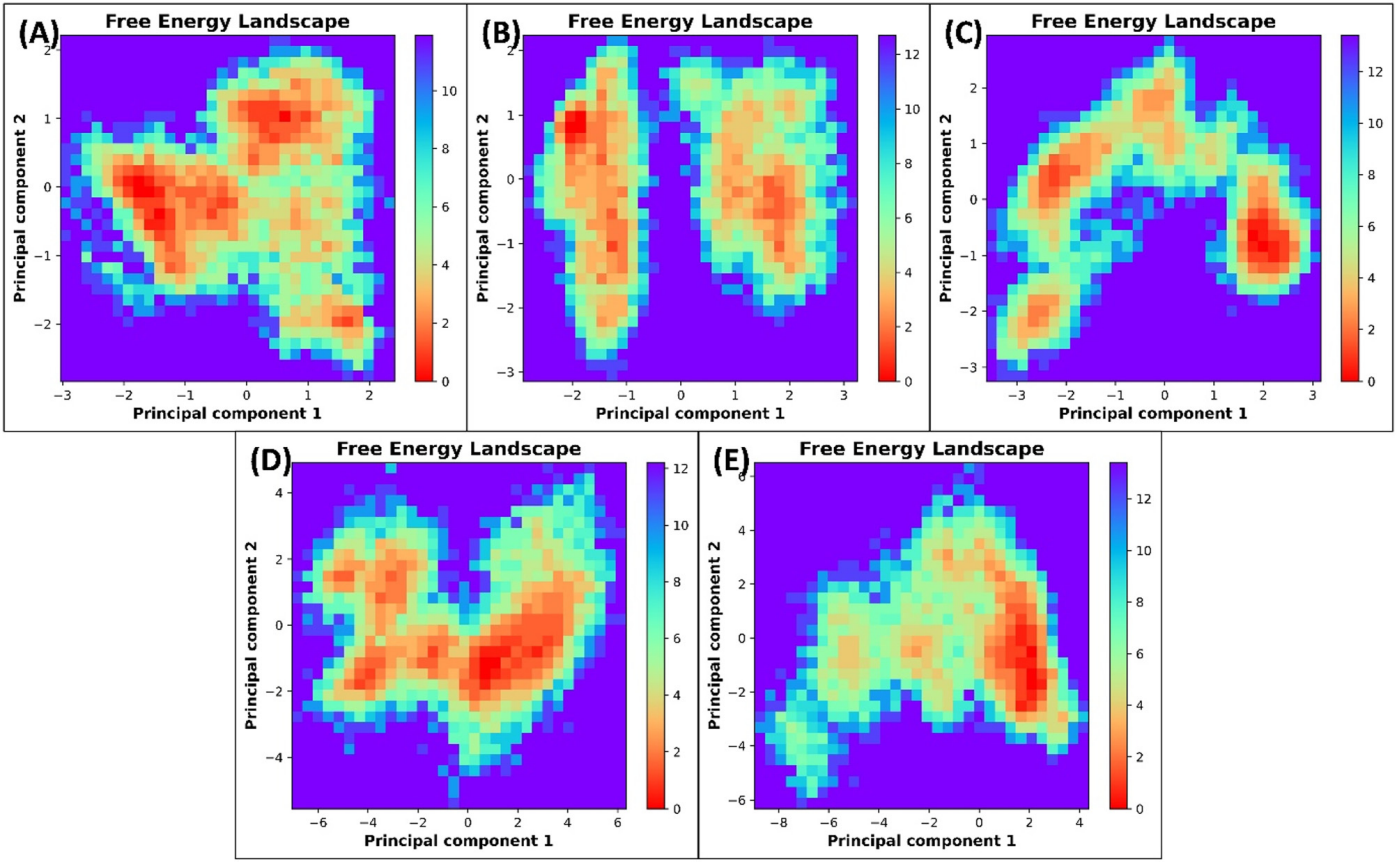
Effect of CA binding on the free energy landscape of HDAC2/H3R. (A) Free energy landscape of apo HDAC2. (B) Free energy landscape of HDAC2-CA complex. (C) Free energy landscape of HDAC2-6EZ complex. (D) Free energy landscape of apo H3R. (E) Free energy landscape of H3R-CA complex.

**Figure 8:**
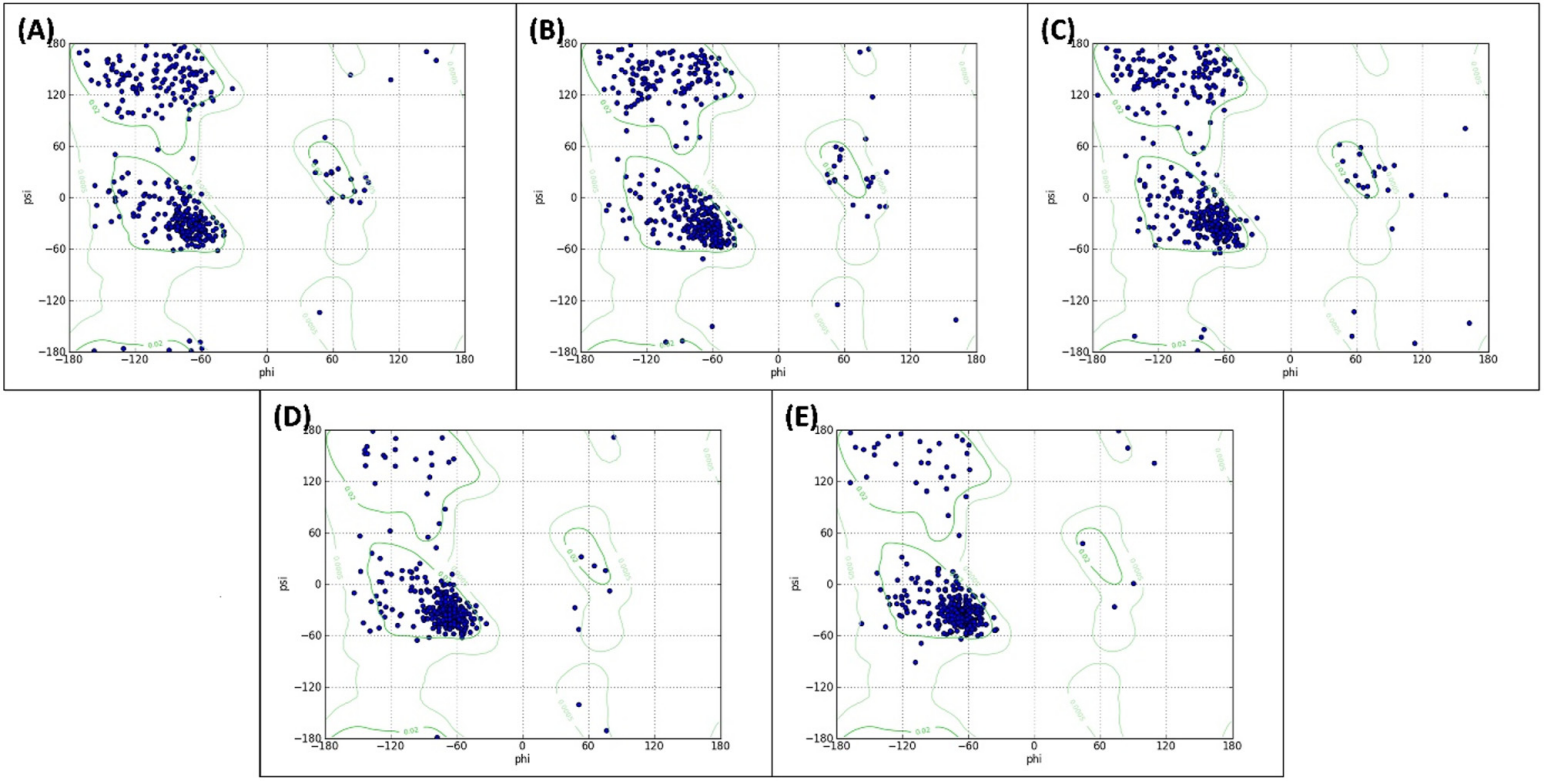
Effect of CA binding on the Ramachandran plot of the energy minimum structure of HDAC2/H3R. (A) Ramachandran plot of the energy minimum structure of apo HDAC2. (B) Ramachandran plot of the energy minimum structure of the HDAC2-CA complex. (C) Ramachandran plot of the energy minimum structure of the HDAC2-6EZ complex. (D) Ramachandran plot of the energy minimum structure of apo H3R. (E) Ramachandran plot of the energy minimum structure of the H3R-CA complex.

The eigenvectors were further utilized to construct the free energy landscape (FEL), as depicted in Figure 7. Notably, all trajectories reached the energy minimum. However, the positions of energy minima differed across the complexes’ landscapes relative to their respective protein-only counterparts. The coordinates of structures corresponding to free energy minima were extracted and further examined using Ramachandran plots (Figure 8). In both apo HDAC2 and HDAC2-6EZ complex, no residues were located within the generously allowed or disallowed regions. However, in the HDAC2-CA complex, only two residues were situated in the generously allowed region, and none were found in the disallowed region. This observation further underscores that the CA interaction did not alter the conformational flexibility of HDAC2. For apo H3R, no residues were located within the generously allowed or disallowed regions. However, for the H3R-CA complex, only one amino acid was found in the disallowed regions. Overall, these structural analyses further validate the stability of the complexes and their positive impact on the structural integrity of HDAC2 and H3R.

#### 3.5.6 Analysis of binding energies between CA and the target protein

**Figure 9:**
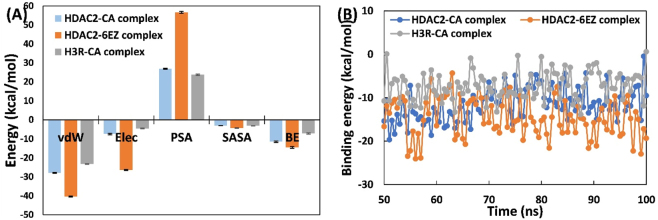
Analysis of binding energies between CA and HDAC2/H3R. (A) MM-PBSA analysis for different energies of HDAC2-CA complex, HDAC2-6EZ complex, and H3R-CA complex. BE, overall binding energy; Elec, electrostatic energy; PSA, polar solvation energy; SASA, SASA energy; vdW, van der Waals energy. (B) Energy per frame of HDAC2-CA complex, HDAC2-6EZ complex, and H3R-CA complex.

The interaction of CA with HDAC2 and H3R was further investigated by evaluating the contributions of various energy components to complex formation. In typical ligand–protein complexes, non-covalent interactions such as hydrophobic, van der Waals, electrostatic, and hydrogen bonds are common and collectively impact the overall interaction. The MM-PBSA binding energies are summarized in Figure 9A. In the CA and HDAC2 complex, van der Waals forces were the primary contributors. Electrostatic energy and SASA energy played minor roles in CA’s interaction with HDAC2. In the complex with HDAC2 involving 6EZ, van der Waals and electrostatic energies were dominant, while SASA energy had minimal impact. When CA binds to H3R, van der Waals energy was the most significant, followed by electrostatic and SASA energies. The polar solvation energy negatively affected the overall interaction in all complexes. The binding energies for CA with HDAC2 and H3R were −11.49 ± 0.37 and −7.20 ± 0.31 kcal/mol, respectively. Notably, the binding energy for the inhibitor (6EZ) with HDAC2 was much higher at −14.54 ± 0.44 kcal/mol compared to CA. These results offer insights into the energetics of ligand-protein interactions. A study of numerous HDAC2 inhibitors reported MM-GBSA ΔG free energies ranging from −7.15 kcal/mol to −20.30 kcal/mol [11].

The MM-PBSA analysis was further utilized to identify the major energy-contributing residues for each interaction, as illustrated in Table 3. In the interaction of CA with HDAC2, highest energy contribution was shown by Met35 followed by Leu144, Pro37, Phe155, Cys156, Gly306, Phe114, Gly305, Ile24, Gly307, Ile40, and His38. In the HDAC2-6EZ complex, key energy-contributing residues were Leu276, Pro34, Tyr308, Phe155, Arg275, His33, Arg39, Arg311, His286, Lys205, Thr309, and Arg315. For the complexation of CA with H3R, Tyr189 was identified as major energy contributing residue followed by Leu37, Tyr91, Trp33, Lys121, Arg381, Trp402, Met41, Phe398, Trp110, Phe193, and Ile88. The energy per frame for 100 trajectory snapshots from 50 ns to 100 ns was also calculated (Figure 9B). The energy per frame remained stable over the tested period and showed no significant fluctuations. For example, the minimum and maximum energies of a single frame of the HDAC2-CA complex are −19.64 and −0.43 kcal/mol, respectively. Similarly, for the H3R-CA complex, one frame’s minimum and maximum energies were −13.84 and 0.60 kcal/mol, respectively. Such energy differences are attributed to the ligand’s movement at the binding site.

**Table 3:** Major energy contributing residues of HDAC2 or H3R for the interaction with CA or 6EZ.

Residues	HDAC2-CA complex		
	Polar energy (kcal/mol)	Apolar energy (kcal/mol)	Total energy (kcal/mol)
Ile24	−0.025	−0.009	−0.380
Met35	0.065	−0.062	−1.258
Pro37	0.015	−0.039	−1.038
His38	−0.177	0.000	−0.270
Ile40	0.206	−0.022	−0.304
Phe114	0.544	−0.101	−0.554
Leu144	0.251	−0.126	−1.084
Phe155	0.615	−0.049	−0.776
Cys156	0.199	−0.028	−0.621
Gly305	−0.44	−0.032	−0.407
Gly306	0.158	−0.066	−0.558
Gly307	−0.07	−0.003	−0.322

Residues	HDAC2-6EZ complex		
	Polar energy (kcal/mol)	Apolar energy (kcal/mol)	Total energy (kcal/mol)
His33	0.432	−0.040	−0.393
Pro34	0.359	−0.226	−1.325
Arg39	−0.454	0.000	−0.316
Phe155	0.414	−0.133	−0.732
Lys205	−0.154	0.000	−0.193
Arg275	1.203	−0.200	−0.575
Leu276	−0.122	−0.241	−1.600
His286	−0.254	0.000	−0.197
Tyr308	1.078	−0.206	−0.824
Thr309	0.434	−0.034	−0.174
Arg311	−0.055	−0.006	−0.266
Arg315	−0.140	0.000	−0.167

Residues	H3R-CA complex		
	Polar energy (kcal/mol)	Apolar energy (kcal/mol)	Total energy (kcal/mol)
Trp33	0.202	−0.073	−0.617
Leu37	0.158	−0.093	−0.883
Met41	0.285	−0.043	−0.341
Ile88	0.047	−0.004	−0.122
Tyr91	1.021	−0.080	−0.782
Trp110	0.406	−0.064	−0.219
Lys121	−0.562	0.000	−0.486
Tyr189	1.157	−0.210	−1.135
Phe193	0.219	−0.039	−0.181
Arg381	−0.53	0.000	−0.405
Phe398	0.344	−0.103	−0.314
Trp402	0.627	−0.067	−0.379

## 4 Discussion

This study employed a comprehensive computational approach to identify CA, a component of *B. vulgaris*, as a potential dual-target agent for ASD therapy, focusing on HDAC2 and H3R. The combination of virtual screening, molecular docking, MD simulations, and ADMET profiling supports CA’s potential. This discussion places these findings within the broader context of existing literature, examining the target rationale, methodological robustness, natural product discovery, mechanistic pathways, and translational implications.

The selection of HDAC2 and H3R is grounded in strong neurobiological precedent. HDAC2 is a key epigenetic regulator with a demonstrated role in neurodevelopment and synaptic function [11]. Its inhibition modulates the expression of genes such as the EAAT2/SLC1A2, which is crucial for glutamate clearance and the prevention of excitotoxicity, a pathway implicated in ASD [50]. Preclinical evidence confirms that HDAC inhibitors can ameliorate autistic-like behaviors, and clinical approval of such compounds for other indications provides a foundational safety precedent. Concurrently, targeting H3R antagonism offers a complementary strategy aimed at cognitive and social domains. The histaminergic system, modulated via presynaptic H3R, influences behaviors relevant to ASD, with antagonists showing efficacy in rodent models of social deficit. This dual-target approach addresses both synaptic homeostasis (via HDAC2/EAAT2) and behavioral modulation (via H3R), representing a mechanistically sophisticated strategy for a heterogeneous disorder [51].

Methodologically, our study employed validated protocols. Docking procedures were rigorously benchmarked, reproducing crystallographic poses of reference inhibitors with low RMSD values [52]. CA demonstrated moderate binding affinities in docking (−7.4 and −7.6 kcal/mol for HDAC2 and H3R, respectively). More insightful were the 100 ns MD simulations and MM-PBSA analyses, which provided a dynamic assessment of stability. The HDAC2-CA complex exhibited a binding free energy of −11.49 ± 0.37 kcal/mol, which was less stable than that of the control inhibitor 6EZ (−14.54 ± 0.44 kcal/mol), consistent with a lower average number of hydrogen bonds. Notably, the complexes exhibited stable RMSD and radius of gyration values, indicating minimal protein distortion upon ligand binding. Residue-level energy contributions, such as Met35 in HDAC2 and Tyr189 in H3R, aligned with known binding pharmacophores, supporting the predicted poses.

The natural product foundation of this work is significant. Screening a library of 26 compounds from *B. vulgaris* leverages the phytochemical diversity of a genus known for bioactive alkaloids and phenolics. While berberine is the most studied neuroprotective component, CA belongs to a different chemical class (a phenylpropanoid ester) and has been reported to protect against oxidative and toxic insults. This positions CA differently from common natural product candidates for ASD (e.g., curcumin, resveratrol), which mainly target oxidative stress and inflammation. Instead, CA’s predicted epigenetic and histaminergic mechanisms offer a new approach for intervention. Additionally, ADMET profiling showed favorable drug-like qualities for CA, including compliance with Lipinski’s and Ghose filters, predicted blood-brain barrier permeability, good water solubility, and no predicted AMES or hERG toxicity. These attributes highlight its potential as a lead compound for CNS drug development.

Mechanistically, the proposed dual action targets complementary ASD-related pathways. Inhibiting HDAC2 is expected to increase EAAT2 expression, thereby promoting glutamate clearance and reducing excitotoxic stress factors associated with repetitive behaviors and synaptic dysfunction. At the same time, H3R antagonism would increase central histaminergic tone, thereby influencing attention, social cognition, and memory [53]. These pathways may act synergistically; for example, maintaining proper glutamate balance could improve the signal-to-noise ratio in histamine-based cognitive processes. This multi-target approach may confer benefits over single-pathway treatments in a complex neurodevelopmental disorder.

Translating these computational predictions requires a clear experimental validation pathway. Essential next steps include *in vitro* enzymatic assays to determine HDAC2 inhibitory potency (IC_50_, *K*_i_) and radioligand binding studies to confirm H3R affinity and functional antagonism. Cellular models, such as patient-derived iPSC neurons or astrocyte co-cultures, could be used to verify EAAT2 upregulation and functional glutamate uptake. Subsequently, behavioral studies in validated ASD rodent models (e.g., valproic acid-exposed or Shank3-deficient mice) are necessary to assess efficacy on social deficits and repetitive behaviors [51]. From a medicinal chemistry perspective, while CA presents a promising lead, optimization may be needed to enhance binding affinity, particularly for Zn^2+^ coordination to HDAC2, and to ensure the metabolic stability of the acetate ester moiety.

Compared to existing methods, this strategy is unique. Most FDA-approved HDAC inhibitors are non-selective, with safety profiles that are not ideal for long-term use in children with ASD. Likewise, current natural product research in ASD mainly focuses on antioxidant polyphenols [54]. CA’s new dual-target mechanism might provide a better-tolerated epigenetic-neurotransmitter modulator. However, limitations need to be recognized. The computational results are predictions and require experimental verification. The moderate binding affinities indicate that CA could serve as a starting point for further optimization rather than as a final clinical candidate.

## 5 Conclusions

ASD manifests early on with deficits in social communication and repetitive sensory-motor behaviors underpinned by a robust genetic basis. Its economic impact persists into adulthood, ranging from mild to severe. Encouragingly, modulation of histone deacetylase (HDAC) activity and antagonism of the histamine H3 receptor (H3R), including FDA-approved drugs, show promise in influencing ASD traits. Using computational modeling and simulation, this study identified potential HDAC2 inhibitors and H3R antagonists. The calculated interaction energies for CA docking to HDAC2 and H3R were −7.4 and −7.6 kcal/mol, respectively. As the lead molecule, CA is expected to attenuate H3R-mediated neuroinflammation and inhibit HDAC2-mediated deacetylation of the EAAT2 gene (SLC1A2). The dual mechanism proposed herein underscores CA’s potential as a therapeutic intervention in treating ASD. These findings provide valuable insights into further exploration and experimental validation of effective treatments for ASD.

## Supplementary Material

Supplementary Material
