# Supplementary material for: Computational approaches for the identification of potential HDAC2 inhibitors and histamine H3 receptor antagonists from Berberis vulgaris: a dual mechanistic approach for autism spectrum disorder treatment
Source: Open Life Sci. 2026 Feb 24;21(1):20251272. doi: 10.1515/biol-2025-1272 (PMC12927461; doi:10.1515/biol-2025-1272)
Supplement: Supplementary file 1 — Supplementary Material [file j_biol-2025-1272_suppl_001.docx]

**Supplementary data**

**Supplementary tables**

| **Supplementary Table S1:** Details of the compounds of *Berberis vulgaris* used in this study. | | | | |
| --- | --- | --- | --- | --- |
| **S. No.** | **Name of phytocompounds** | **PubChem CID** | **Formula** | **MW** |
| 1 | Cinnamyl acetate | 5282110 | C_11_H_12_O_2_ | 176.21 |
| 2 | trans-sesquisabinene hydrate | 6428444 | C_15_H_26_O | 222.37 |
| 3 | Ylangene | 20055075 | C_15_H_24_ | 204.35 |
| 4 | Sesquicineole | 341779 | C_15_H_26_O | 222.37 |
| 5 | 2-Hydroxycinnamic acid | 637540 | C_9_H_8_O_3_ | 164.16 |
| 6 | Alpha-longipinene | 12311396 | C_15_H_24_ | 204.35 |
| 7 | Guaiol | 227829 | C_15_H_26_O | 222.37 |
| 8 | alpha-curcumene | 442360 | C_15_H_22_ | 202.34 |
| 9 | alpha-guaiene | 5317844 | C_15_H_24_ | 204.35 |
| 10 | Guaia-1-10-11-diene | 520826 | C_15_H_24_ | 204.35 |
| 11 | Berberine | 2353 | C_20_H_18_NO_4_^+^ | 336.36 |
| 12 | Cinnamyl alcohol | 5315892 | C_9_H_10_O | 134.18 |
| 13 | Columbamine | 72310 | C_20_H_20_NO_4_^+^ | 338.38 |
| 14 | 7-10-pentadecadiynoic acid | 30942 | C_15_H_22_O_2_ | 234.33 |
| 15 | Stigmasterol | 5280794 | C_29_H_48_O | 412.69 |
| 16 | Methyl-5-7-hexadecadiynoate | 14957560 | C_17_H_26_O_2_ | 262.39 |
| 17 | Oxyberberine | 11066 | C_20_H_17_NO_5_ | 351.35 |
| 18 | Lambertine | 10217 | C_20_H_19_NO_4_ | 337.37 |
| 19 | Mesitylene | 7947 | C_9_H_12_ | 120.19 |
| 20 | Palmatine | 19009 | C_21_H_22_NO_4_^+^ | 352.4 |
| 21 | Phenylethyl alcohol | 6054 | C_8_H_10_O | 122.16 |
| 22 | Guaiacol | 460 | C_7_H_8_O_2_ | 124.14 |
| 23 | 2-Methoxy-4-vinylphenol | 332 | C_9_H_10_O_2_ | 150.17 |
| 24 | Stigmasterolglucoside | 6602508 | C_35_H_58_O_6_ | 574.83 |
| 25 | 2-pentenoic acid | 638122 | C_5_H_8_O_2_ | 100.12 |
| 26 | 3-ethoxypropionaldehyde | 17755 | C_5_H_10_O_2_ | 102.13 |
|  | MW is molecular weight. | | | |

| **Supplementary Table S2:** Prediction of ADME properties of the compounds of *Berberis vulgaris* using the pkCSM webserver. | | | | | | | | |
| --- | --- | --- | --- | --- | --- | --- | --- | --- |
| **Name of phytocompounds** | **Absorption** | | **Distribution** | | **Metabolism** | | **Excretion** | |
|  | **Intestinal absorption** | **Water solubility (mg/ml)** | **VDss (human) (log L/kg)** | **Fraction unbound (human)** | **CYP3A4 substrate** | **CYP3A4 inhibitor** | **Total Clearance** | **Renal OCT2 substrate** |
| Cinnamyl acetate | 96.71 | 1.06 | 0.14 | 0.26 | No | Yes | 0.377 | No |
| trans-sesquisabinene hydrate | 93.66 | 0.01 | 0.55 | 0.26 | No | No | 1.112 | No |
| Ylangene | 96.22 | 0.00 | 0.81 | 0.12 | Yes | Yes | 0.95 | No |
| Sesquicineole | 94.41 | 0.00 | 0.68 | 0.31 | No | No | 1.129 | No |
| 2-Hydroxycinnamic acid | 93.49 | 0.63 | -1.19 | 0.42 | No | No | 0.736 | No |
| Alpha-longipinene | 95.79 | 0.00 | 0.89 | 0.27 | No | No | 0.863 | No |
| Guaiol | 94.00 | 0.02 | 0.48 | 0.35 | No | No | 1.077 | No |
| alpha-curcumene | 93.29 | 0.00 | 1.09 | 0.02 | Yes | Yes | 1.511 | No |
| alpha-guaiene | 95.51 | 0.00 | 0.68 | 0.26 | No | No | 1.219 | No |
| Guaia-1-10-11-diene | 93.43 | 0.00 | 0.68 | 0.18 | Yes | No | 1.216 | No |
| Berberine | 97.15 | 0.04 | 0.58 | 0.26 | Yes | Yes | 1.27 | No |
| Cinnamyl alcohol | 92.67 | 1.91 | 0.30 | 0.34 | No | Yes | 0.253 | No |
| Columbamine | 94.33 | 0.05 | 0.54 | 0.23 | Yes | Yes | 1.224 | No |
| 7-10-pentadecadiynoic acid | 95.97 | 0.01 | -0.74 | 0.17 | Yes | No | 1.698 | No |
| Stigmasterol | 94.97 | 0.00 | 0.18 | 0.00 | Yes | No | 0.618 | No |
| Methyl-5-7-hexadecadiynoate | 95.93 | 0.00 | 0.18 | 0.11 | Yes | Yes | 1.794 | No |
| Oxyberberine | 100.00 | 0.02 | -0.03 | 0.17 | Yes | Yes | 0.121 | Yes |
| Lambertine | 95.29 | 0.02 | 0.38 | 0.03 | Yes | Yes | 0.28 | Yes |
| Mesitylene | 95.51 | 0.16 | 0.36 | 0.34 | No | No | 0.264 | No |
| Palmatine | 97.08 | 0.02 | 0.64 | 0.25 | Yes | Yes | 1.246 | No |
| Phenylethyl alcohol | 88.07 | 7.74 | 0.20 | 0.43 | No | Yes | 0.325 | No |
| Guaiacol | 93.37 | 6.76 | 0.17 | 0.50 | No | No | 0.219 | No |
| 2-Methoxy-4-vinylphenol | 91.97 | 1.65 | 0.12 | 0.32 | No | Yes | 0.233 | No |
| Stigmasterolglucoside | 78.90 | 0.01 | -1.16 | 0.08 | Yes | No | 0.674 | No |
| 2-pentenoic acid | 90.49 | 21.85 | -0.86 | 0.65 | No | No | 0.916 | No |
| 3-ethoxypropionaldehyde | 100.00 | 113.02 | -0.16 | 0.73 | No | No | 0.795 | No |
| VDss is volume of distribution, Intestinal absorption is for human. | | | | | | | | |

| **Supplementary Table S3:** Prediction of drug likeness and pharmacokinetics of the compounds of *Berberis vulgaris* using the SwissADME webserver. | | | | | |
| --- | --- | --- | --- | --- | --- |
| **Name of phytocompounds** | **Drug likeness** | | **Pharmacokinetics** | | |
|  | **No. of Lipinski violations** | **No. of Ghose violations** | **GI absorption** | **BBB permeant** | **PGP substrate** |
| Cinnamyl acetate | 0.00 | 0.00 | High | Yes | No |
| trans-sesquisabinene hydrate | 0.00 | 0.00 | High | Yes | No |
| Ylangene | 1.00 | 0.00 | Low | Yes | No |
| Sesquicineole | 0.00 | 0.00 | High | Yes | No |
| 2-Hydroxycinnamic acid | 0.00 | 0.00 | High | Yes | No |
| Alpha-longipinene | 1.00 | 0.00 | Low | No | No |
| Guaiol | 0.00 | 0.00 | High | Yes | No |
| alpha-curcumene | 1.00 | 0.00 | Low | No | No |
| alpha-guaiene | 1.00 | 0.00 | Low | No | No |
| Guaia-1-10-11-diene | 1.00 | 0.00 | Low | No | No |
| Berberine | 0.00 | 0.00 | High | Yes | Yes |
| Cinnamyl alcohol | 0.00 | 1.00 | High | Yes | No |
| Columbamine | 0.00 | 0.00 | High | Yes | Yes |
| 7-10-pentadecadiynoic acid | 0.00 | 0.00 | High | Yes | No |
| Stigmasterol | 1.00 | 3.00 | Low | No | No |
| Methyl-5-7-hexadecadiynoate | 1.00 | 0.00 | High | Yes | No |
| Oxyberberine | 0.00 | 0.00 | High | Yes | Yes |
| Lambertine | 0.00 | 0.00 | High | Yes | Yes |
| Mesitylene | 1.00 | 1.00 | Low | Yes | No |
| Palmatine | 0.00 | 0.00 | High | Yes | Yes |
| Phenylethyl alcohol | 0.00 | 3.00 | High | Yes | No |
| Guaiacol | 0.00 | 3.00 | High | Yes | No |
| 2-Methoxy-4-vinylphenol | 0.00 | 1.00 | High | Yes | No |
| Stigmasterolglucoside | 1.00 | 4.00 | High | No | Yes |
| 2-pentenoic acid | 0.00 | 3.00 | High | Yes | No |
| 3-ethoxypropionaldehyde | 0.00 | 3.00 | High | Yes | No |
| GI absorption is gastrointestinal absorption, BBB is blood brain barrier permeant, PGP substrate is P-glycoprotein substrate. | | | | | |

**Supplementary figure**

**Supplementary Figure S1:** (A) Active site of HDAC2. The active site region is shown as yellow surface residues. (B) Active site of residues of HDAC2. Active site residues are shown as yellow sticks with labels. HDAC2 is shown in grey ribbons. (C) Zn metal coordination of HDAC2. (D) Ligand-binding regions of H3R.
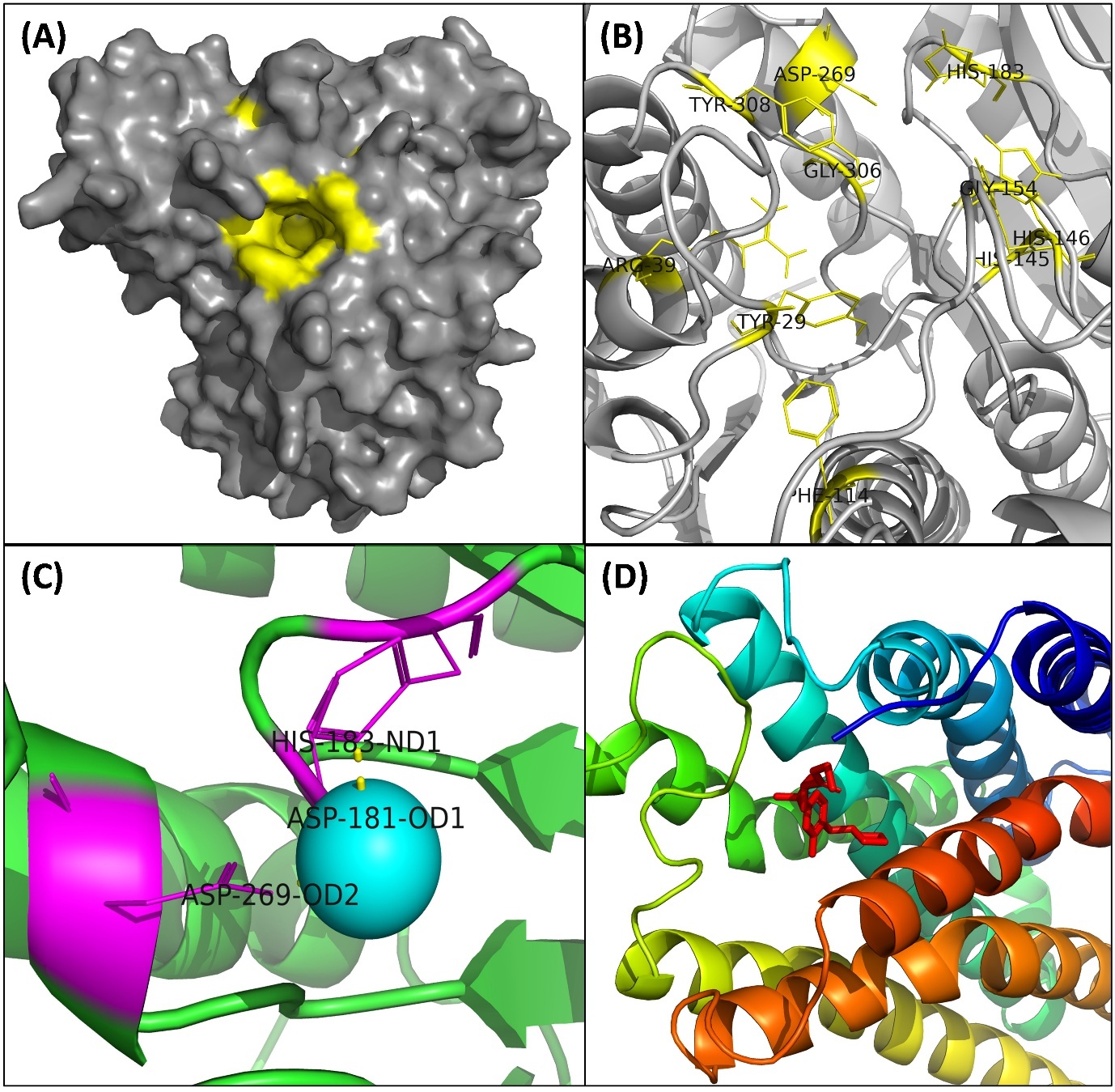


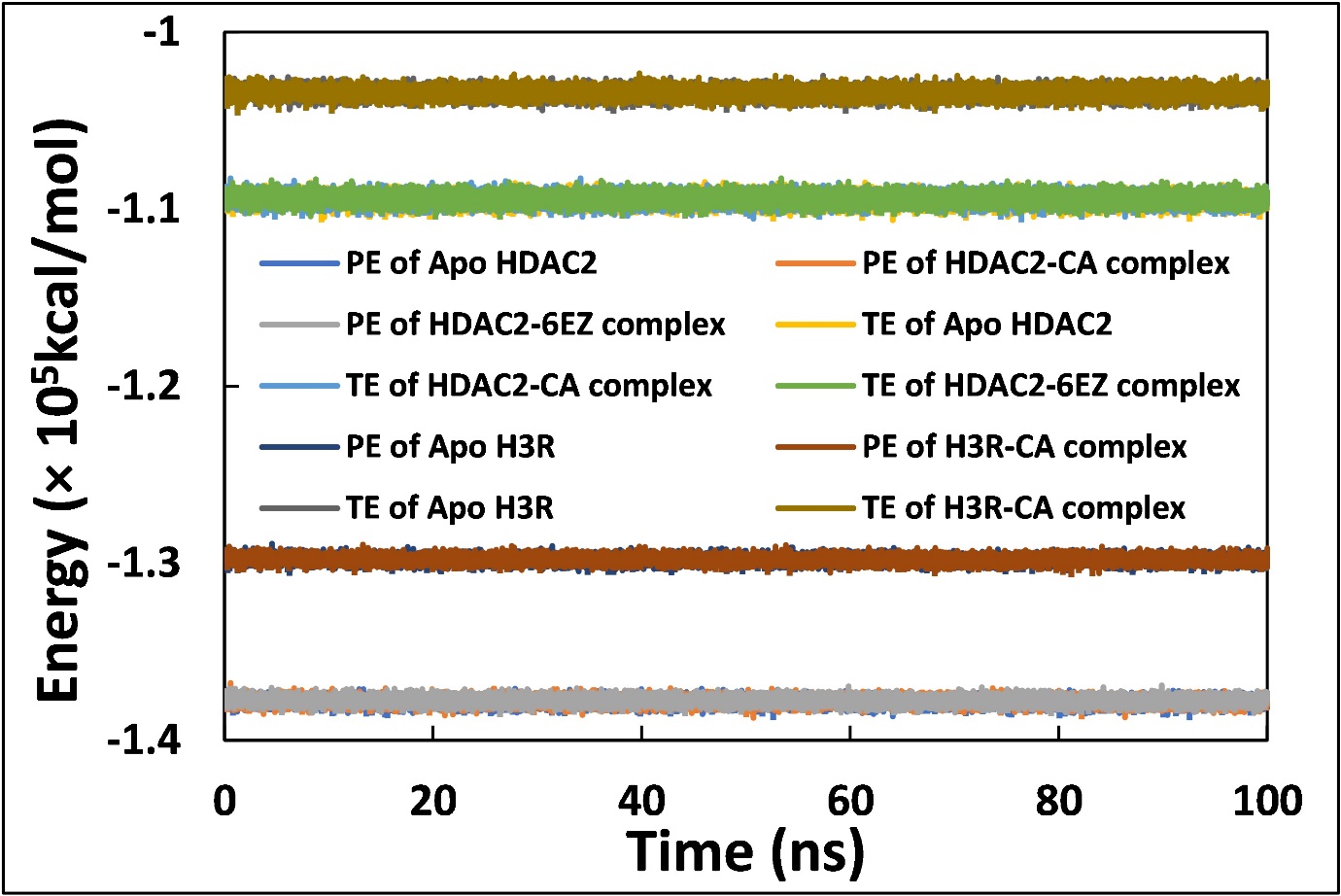


**Supplementary Figure S2**: Total energy (TE) and potential energy (PE) of apo HDAC2, HDAC2-CA complex, HDAC2-6EZ complex, apo H3R, and H3R-CA complex over simulation time.
